# The first multi-tissue genome-scale metabolic model of a woody plant highlights suberin biosynthesis pathways in *Quercus suber*

**DOI:** 10.1371/journal.pcbi.1011499

**Published:** 2023-09-20

**Authors:** Emanuel Cunha, Miguel Silva, Inês Chaves, Huseyin Demirci, Davide Rafael Lagoa, Diogo Lima, Miguel Rocha, Isabel Rocha, Oscar Dias

**Affiliations:** 1 Centre of Biological Engineering, Universidade do Minho, Braga, Portugal; 2 Instituto de Tecnologia Química e Biológica António Xavier, Universidade Nova de Lisboa, Avenida da República, Quinta do Marquês, Oeiras, Portugal; 3 iBET, Instituto de Biologia Experimental e Tecnológica, Oeiras, Portugal; 4 SnT/University of Luxembourg, Luxembourg; 5 LABBELS–Associate Laboratory, Braga, Guimarães, Portugal; Christian Albrechts Universitat zu Kiel, GERMANY

## Abstract

Over the last decade, genome-scale metabolic models have been increasingly used to study plant metabolic behaviour at the tissue and multi-tissue level under different environmental conditions. *Quercus suber*, also known as the cork oak tree, is one of the most important forest communities of the Mediterranean/Iberian region. In this work, we present the genome-scale metabolic model of the *Q*. *suber* (iEC7871). The metabolic model comprises 7871 genes, 6231 reactions, and 6481 metabolites across eight compartments. Transcriptomics data was integrated into the model to obtain tissue-specific models for the leaf, inner bark, and phellogen, with specific biomass compositions. The tissue-specific models were merged into a diel multi-tissue metabolic model to predict interactions among the three tissues at the light and dark phases. The metabolic models were also used to analyse the pathways associated with the synthesis of suberin monomers, namely the acyl-lipids, phenylpropanoids, isoprenoids, and flavonoids production. The models developed in this work provide a systematic overview of the metabolism of *Q*. *suber*, including its secondary metabolism pathways and cork formation.

## Author summary

Our study presents a comprehensive metabolic model of Quercus suber, also known as the cork oak tree, which is a keystone species of the Mediterranean forests. We used a genome-scale approach to build a model (iEC7871) that integrates transcriptomics data and accounts for the metabolic interactions among different tissues of the tree. Specifically, we derived tissue-specific models for the leaf, inner bark, and phellogen, which are the main tissues involved in cork production. We then combined these models into a diel multi-tissue model that simulates the metabolic behaviour of the tree under light and dark conditions. Our model includes the pathways associated with the biosynthesis of suberin monomers, which are the main components of cork. By analysing these pathways, we identified potential targets for improving cork quality and yield in Q. suber. Our work provides a valuable resource for studying the metabolism of cork oak trees and highlights the importance of integrating multi-omics data to build predictive models of plant metabolism.

## 1. Introduction

The cork oak, *Quercus suber* L., is a characteristic tree of the Mediterranean/Iberian landscape ecosystem. The tree forms a thick bark of cork (phellem) containing high levels of aliphatic and aromatic suberin, extractives (waxes and tannins), and polysaccharides [1]. Cork properties are so unique that this material is used in diverse applications from the common bottle-stoppers to spacecrafts built by the National Aeronautics and Space Administration and European Space Agency, providing insulation solutions to deal with extreme conditions. The aliphatic suberin component has particular interest since it provides waterproof, light, elastic, and fire-retardant properties to cork [2,3]. Only the outer bark is separated from the trunk during the harvest, which enables regeneration and allows using the tree as a renewable biological resource. Cork extraction can continue in the same tree for more than a century every 9 years. A 234-year-old cork oak tree, debarked 20 times, is now a living icon being elected European tree of the year 2018 (https://www.treeoftheyear.org/Previous-Years/2018).

Phellem is produced by the phellogen (cork cambium), involving the proliferation of phellogen derivates cells which undergo differentiation into cork cells through cell expansion, deposition of suberin and waxes, and an irreversible senescence program ending with cell death. The first debarking occurs when the tree has 18–25 years old. This first harvested cork, known as “virgin” cork, produced from the original phellogen, yields poor-quality cork. The original phellogen is then replaced by a traumatic phellogen that proliferates to originate a new cork layer. From the third debarking onwards, a higher quality cork with high economic value is obtained—reproduction cork (or “amadia”) [4]. Cork growth and quality are dependent on the genome of each tree, but it is also strongly dependent on environmental conditions, such as water availability, temperature, pests, and diseases.

The cork oak tree represents one of the most relevant broadleaved forest resources in the Mediterranean basin (Portugal, Spain, Algeria, Morocco, France, Italy, and Tunisia), having a substantial socio-economic and ecological impact in these countries. This species can generally live over 200 years, which implies a capacity to bear with biotic stresses like bacteria, fungi and insects, as well as abiotic stresses like drought, floods and fires [5,6].

Due to its economic interest in Portugal, a national consortium was created to first sequence the transcriptome [7] and recently to fully sequence the genome of *Quercus suber* [8]. The genome sequence’s availability allows the application of emergent systems biology tools, like Genome-scale metabolic (GSM) models.

Genome-scale metabolic models aim at depicting the whole metabolic network of an organism. Such models have been widely used for metabolic engineering purposes, mainly with prokaryotes and yeasts. Among other applications, GSM models can be used to analyse an organism’s metabolic traits in different environmental and genetic conditions, including the effect of gene knock-outs and over/under-expression [9]. High-quality models have proven to accurately predict complex organisms’ metabolic behaviour in diverse areas of knowledge, from biotechnological or environmental to medical applications [10–14]. Genome-scale modelling in plants is much more challenging than in prokaryotes. The struggle starts in the annotation of the complex genomic content, in which the function of a significant number of genes is still unknown. Also, subcellular compartmentalization, tissue differentiation, and interactions between tissues are complex in plants. In the last decade, the number of published GSM models of plants has increased considerably, focusing on model organisms, like *Arabidopsis thaliana* [15,16], *Zea mays* (maize) [17,18], and *Oryza sativa* (rice) [19,20]. In 2020, a metabolic model of *Populus trichocarpa* (black cottonwood), was published [21]. This manuscript highlighted the impact of single nucleotide polymorphism in carbon and energy partitioning, and lignin biosynthesis. Considering the importance of plants in terms of nutrition, biofuels, and their capability to produce a variety of secondary metabolites, it is not surprising that studies for plant genome-scale model reconstruction will increase, parallel to the growth in the number of sequenced species. A GSM model for *Q*. *suber* would be useful to provide insights into the metabolic behaviour of this species in its different tissues through different development stages, as well as the tree’s response to stress conditions.

This paper describes the reconstruction of iEC7871, a genome-scale metabolic model for the cork oak tree. Besides the generic GSM, we present tissue-specific models and a multi-tissue metabolic model that can be used to study the metabolic behaviour of *Q*. *suber* at the multi-tissue level.

## 2. Results

### 2.1. Model properties

A GSM model for *Q*. *suber* based on an up-to-date genome annotation was reconstructed in this work.

A Basic Local Alignment Search Tool (BLAST) [22] search against the UniProtKB/Swiss-Prot [23] database allowed to identify similarity results for 47,199 out of the 59,614 genes available in the genome [8]. A second BLAST search against UniProtKB/TrEMBL allowed obtaining hits for 12,415 genes, while 590 had no results within the defined BLAST parameters. Based on the results of these homology searches, the genome of *Q*. *suber* was functionally annotated using the automatic workflow available in *merlin*. A detailed analysis of the genome annotation is available in S1 File.

The metabolic model is mass balanced and can predict growth in phototrophic and heterotrophic conditions. These conditions were defined by setting the photon and *CO*_2_ (phototrophic), or sucrose (heterotrophic) as the sole energy and carbon sources, respectively. Additionally, the model requires *H*_2_*O*, *NH*_3_ or *HNO*_3_, *H*_3_*PO*_4_, *H*_2_*SO*_4_, *Fe*^2+^, and *Mg*^2+^ to produce biomass.

The general properties of the iEC7871 and five published plant models–*A*. *thaliana* [18,24], *Z*. *mays* [17,18], and *Glycine max* (soybean) [25]—are presented in Table 1. Seaver *et al*. (2015) [18] developed full and evidence-based models for *A*. *thaliana* and *Z*. *mays*. The full models were developed by integrating data retrieved from KEGG, BioCyC databases, and published GSM models. These models were manually refined by evaluating the gene-reaction associations, originating evidence-based models.

**Table 1:** General properties (genes, reactions, metabolites, and compartments) of the *Q*. *suber* model, and other five published plant GSM models. Only generic models were considered here.

	*Q*. *suber* (this work)	*A*. *thaliana* (2013) [24]	*A*. *thaliana* (2015) [18]ƚ	*Z*. *mays* (2011) [17]	*Z*. *mays* (2015) [18]ƚ	*G*. *max* (2019) [25]
**Genes**	7,871	1,475	2,400 (5,184)	1,563	3,413 (13,279)	6,127
**Reactions**	6,232	1,895	2,801 (6,399)	1,970	2,629 (6,458)	2,984
**Metabolites**	6,482	1,761	2,864 (6,236)	2,129	2,634 (6,250)	2,814
**Compartments**	8	6	10	6	10	5

ƚ Seaver et *al*. (2015) [18] generated full and evidence-based models for *A*. *thaliana* and *Zea mays*. Data for the full models are shown in parentheses.

The iEC7871 comprises 7,871 genes, 6,232 reactions (including enzymatic, spontaneous, and transport), of which 3,260 are blocked. Additionally, there are 708 exchange reactions, and 6,482 metabolites distributed across eight subcellular compartments–the cytoplasm, mitochondria, plastid, endoplasmic reticulum, Golgi apparatus, vacuole, peroxisome, and the extracellular space. Most of the metabolic reactions included in the model were derived from homology searches (4,527 out of 6,232 reactions), while 987 transport reactions were automatically added by TranSyT. During the manual curation stage, 638 reactions were manually added to the model, including 293 KEGG reactions not identified when generating the draft network either due to missing and wrong gene annotations, as well as a lack of information regarding some KEGG reactions (e.g., missing and incomplete EC numbers, reactions not well characterized). From the 264 transport reactions manually added, 225 have no gene associated. These included several simple diffusion transport reactions for molecules like oxygen across the different compartments, as well as transporters required to ensure network connectivity. In addition, *merlin* automatically retrieves spontaneous reactions from KEGG, of which 80 were kept in the model. The source of all reactions is available in Table N in S2 File.

Only the full models of *A*. *thaliana* and *Z*. *mays* models developed by Seaver *et al*. (2015) [18] have a similar number of reactions and metabolites as the iEC7871. The reactions and metabolites included in the iEC7871 were compared with the ones available in the evidence-based models developed by Seaver *et al*. (2015) [18]. As these models are available with ModelSEED [26] identifiers, we converted all reaction and metabolite identifiers into MetaNetX [27] identifiers to allow the comparison. Besides manually added reactions, only five reactions did not have a match (“R11261,”, “R02889”, “R11307”, “rxn22163”, and “rxn37218”). All metabolites with KEGG or ModelSEED IDs were converted into MetaNetX IDs successfully. In addition, we compare the iEC7871 with the models developed by Chung *et al*. (2013) [24] and Saha *et al*. (2011) [17], which provide KEGG identifiers (Tables B-G in S2 File).

**Fig 1:**
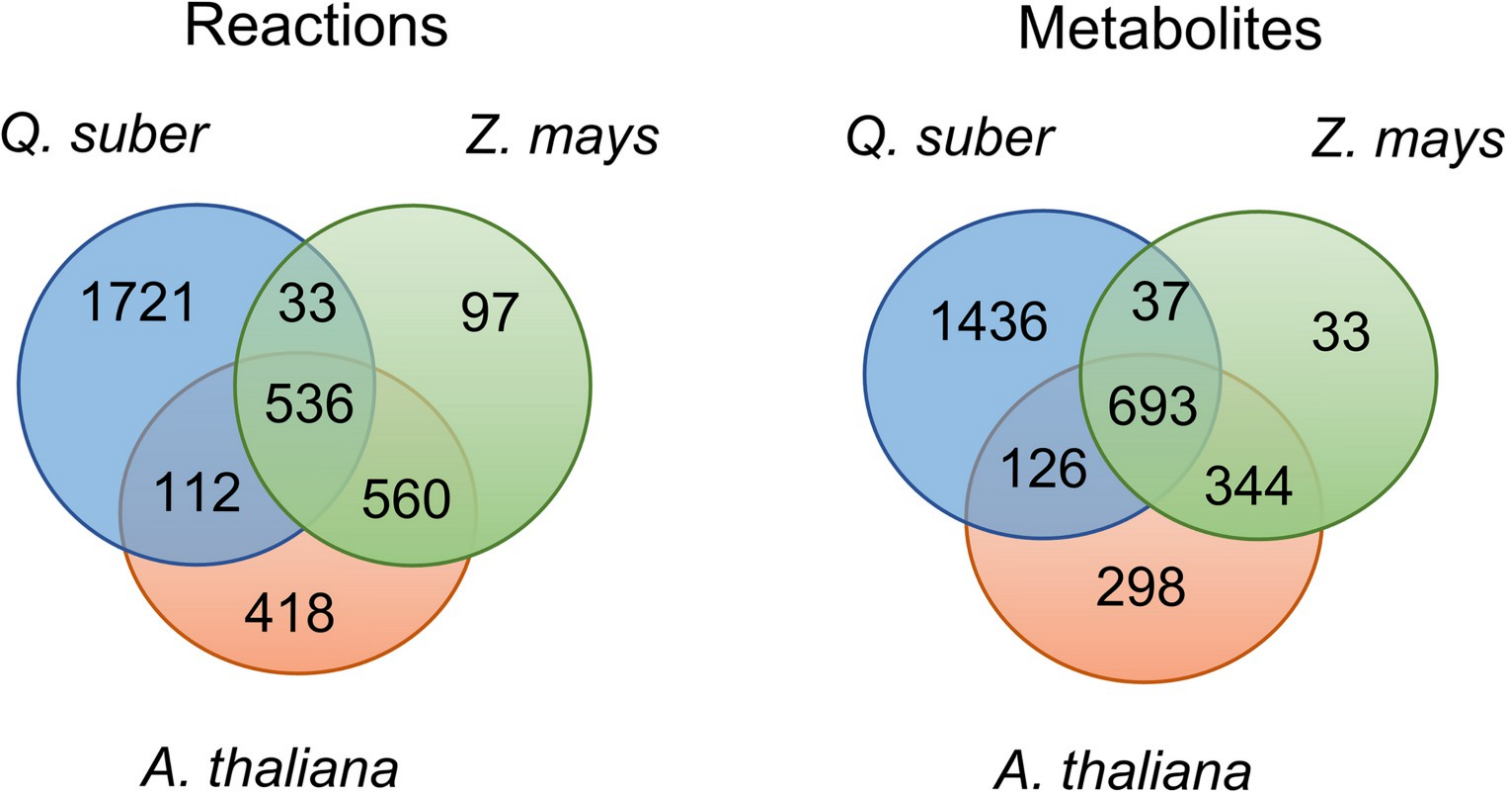
Venn diagram of reactions (left) and metabolites (right) included in the *Q*. *suber*, *A*. *thaliana*, and *Z*. *mays* models.

A total of 3,477 unique reactions and 2,967 unique metabolites were identified across the three models. The iEC7871 includes 1,721 reactions that are not present in the other two—Fig 1. It has 33 reactions in common only with the Maize model and 112 only with the *A*. *thaliana* model. The maize and *A*. *thaliana* models share 560 reactions, and 536 were found in the three models. The metabolites analysis shows similar behaviour.

The reactions available only in the iEC7871 (called unique reactions in the following) were further analysed to assess the annotation of the genes and pathways associated with them (Table H in S2 File). In agreement with the remaining genome annotation, 82% of the genes encoding enzymes that catalyse these reactions were annotated based on *A*. *thaliana* genes’ annotations. The remaining 18% were annotated by gene records of organisms not accounted for in the annotation workflow.

The KEGG pathways with more unique reactions are presented in Table 2. The 28 pathways with at least 20 unique reactions can be divided into eight of the 13 main areas of the KEGG metabolism: 11 pathways representing “Lipid metabolism”, 4 “aminoacid metabolism”, 3 “Xenobiotics biodegradation metabolism”, 3 “Metabolism of cofactors and vitamins” and “Biosynthesis of other secondary metabolites”, 4 the “Metabolism of terpenoids and polyketides”, 2 the “Nucleotide metabolism”, and 1 the “Genetic Information Processing”. Spontaneous reactions and reactions not associated with any pathway were not considered in this analysis.

**Table 2:** Number of reactions in the iEC7871 not identified in the *A*. *thaliana* and *Z*. *mays* [18] models for each pathway (so-called unique reactions). Only pathways with more than 20 unique reactions were included in this table. The complete table is available in Table H in S2 File. The KEGG pathways were organized according to the main areas of KEGG metabolism: 1- Xenobiotics biodegradation and metabolism; 2- Lipid metabolism; 3- Metabolism of cofactors and vitamins; 4- Amino acid metabolism and Metabolism of other amino acids; 5- Metabolism of terpenoids and polyketides; 6- Biosynthesis of other secondary metabolites; 7- Nucleotide metabolism; 8- Genetic Information Processing.

	KEGG Pathway	Number of unique reactions
**2**	Fatty acid metabolism	85
**1**	Metabolism of xenobiotics by cytochrome P450	56
**2**	Fatty acid biosynthesis	51
**7**	Pyrimidine metabolism	46
**2**	Glycerophospholipid metabolism	41
**2**	Steroid biosynthesis	41
**7**	Purine metabolism	41
**6**	Flavonoid biosynthesis	40
**4**	Tryptophan metabolism	40
**2**	Steroid hormone biosynthesis	39
**5**	Carotenoid biosynthesis	37
**5**	Ubiquinone and other terpenoid-quinone biosynthesis	29
**4**	Tyrosine metabolism	29
**5**	Brassinosteroid biosynthesis	28
**4**	Cysteine and methionine metabolism	27
**1**	Drug metabolism—other enzymes	27
**3**	Porphyrin and chlorophyll metabolism	27
**2**	Fatty acid degradation	27
**1**	Drug metabolism—cytochrome P450	25
**6**	Phenylpropanoid biosynthesis	24
**2**	Inositol phosphate metabolism	24
**4**	Arginine and proline metabolism	22
**2**	Biosynthesis of unsaturated fatty acids	21
**8**	Aminoacyl-tRNA biosynthesis	20
**5**	Diterpenoid biosynthesis	20
**2**	Sphingolipid metabolism	20
**2**	Arachidonic acid metabolism	20
**2**	Fatty acid elongation	20

The “unique reactions” identified are associated with 125 different KEGG pathways, from which 36 had five or fewer unique reactions (Table H in S2 File). “Fatty acid metabolism”, “Metabolism of xenobiotics by cytochrome P450”, “Fatty acid biosynthesis”, and “Pyrimidine metabolism” are the pathways with more unique reactions, having 46 to 85 reactions unavailable in the maize and *A*. *thaliana* models.

Several of these reactions might be related to species-specific reactions, reflecting the cork oak’s uniqueness and differences from non-woody plants. Although the reactions are cork oak specific, these belong to pathways already present in other species. Some unique reactions are associated with pathways essential for trees and belong to the secondary metabolism responsible for wood and cork production, such as “Phenylpropanoid biosynthesis”, “Flavonoid biosynthesis”, “Fatty acid biosynthesis”, “Metabolism of xenobiotics by cytochrome P450”, and “Cutin, suberine and wax biosynthesis”.

### 2.2. Flux scanning based on enforced objective flux

Suberin is one of the major components of the phellogen. It was reported that reproduction cork presents a higher percentage of suberin than virgin cork. Thus, the flux scanning based on enforced objective flux (FSEOF) method [28] was applied to the generic model. This method allowed identifying 147 reactions whose flux increased with enforced suberin production. The targets identified were subject to overexpression simulation and analysis using minimization of metabolic adjustment (MOMA) [29]. The target reactions were ranked using an evaluation function (f_ph_). This procedure allowed identifying 52 reactions with cumulative effect on suberin and biomass production. The top 10 enzymatic reaction targets and associated metabolic pathways are listed in Table 3.

**Table 3:** List of top ten enzymatic reaction targets ranked by the evaluation function for increased suberin production.

Rank	Reaction Identifier	Enzyme	Metabolic pathway	f_ph_
**1**	R00842__cyto	Glycerol-3-phosphate dehydrogenase	Glycerophospholipid metabolism	1.00
**2**	R09452__e_r_	Long-chain fatty acid omega-monooxygenase	Cutin, suberine and wax biosynthesis	0.99
**3**	R03366__cyto	Caffeate O-methyltransferase	Phenylpropanoid biosynthesis	0.99
**4**	R07826__e_r_	Coumarate 3-hydroxylase	Phenylpropanoid biosynthesis	0.99
**5**	R10092__cyto	Carbonic anhydrase	Nitrogen metabolism	0.97
**6**	R08551__e_r_	Cytochrome P-450 reductase	Cytochrome P-450	0.97
**7**	R00742__plst	Acetyl-CoA carboxylase	Fatty acid metabolism	0.94
**8**	R01845__plst	Sedoheptulose-1,7-bisphosphatase	Carbon fixation	0.93
**9**	R08163__plst	Fatty acyl-ACP thioesterase A	Fatty acid metabolism	0.93
**10**	R00742__plst	Acetyl-CoA carboxylase	Fatty acid metabolism	0.93

The analysis of the top reaction targets highlighted the importance of specific reactions and metabolic pathways that affect suberin production. The reaction “R00842__cyto”, catalysed by glycerol-3-phosphate dehydrogenase, was identified as the reaction with the most impact on suberin biosynthesis. This enzyme is responsible for the conversion between glycerone phosphate and glycerol-3-phosphate, a component of suberin. Similarly, caffeate O-methyltransferase and p-coumarate 3-hydroxylase, involved in the biosynthesis of another suberin component (ferulate) were recognized as targets. This analysis also identified several reactions associated with fatty acid metabolism (e.g., long-chain fatty acid omega-monooxygenase, acetyl-CoA carboxylase, and fatty acyl-ACP thioesterase A), as well as the cytochrome P-450 reductase. Several other reactions associated with fatty acid biosynthesis, elongation, and modification were identified (Table A in S4 File).

### 2.3. Tissue-specific models

Transcriptomics data were integrated into the generic model using *troppo* [30] to obtain tissue-specific models. Leaf, Inner bark, Reproduction Phellogen, and Virgin Phellogen were selected because they influence tree growth and cork production. The data for the leaf and inner bark was retrieved from the *Q*. *suber* genome sequencing project, while the phellogen data was obtained in a genome-wide transcriptomic analysis of the phellem and xylem of *Q*. *suber* [31].

**Fig 2:**
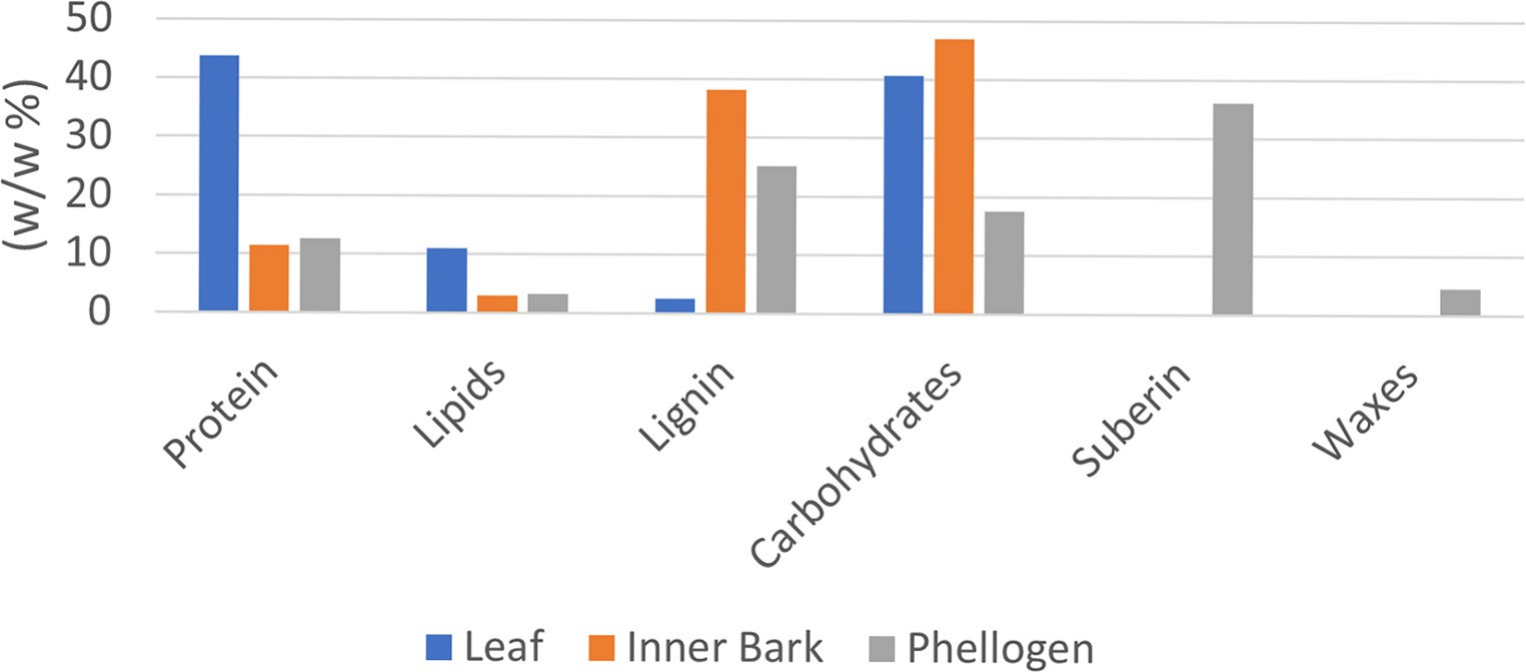
The biomass compositions of the leaf, inner bark, and phellogen were determined using data retrieved from published plant GSM models and available experimental data. DNA, RNA, and cofactors were omitted from the plot as their contribution to the biomass is very low (<2%) in all tissues. The complete biomass composition and the respective data sources are detailed in S3 File.

Different biomass formulations for each tissue were determined (Fig 2), according to experimental data available in the literature and other plant GSM models. The formulation of biomass components using other organisms’ data was based on their phylogenetic (S1 File) and metabolic (e.g., C3 vs C4 photosynthesis) proximity to *Q*. *suber*. The detailed biomass composition for each tissue is available in S3 File.

As described in the Materials and Methods section, the biomass was formulated by creating “e-Metabolites”, representing the macromolecular composition of each tissue. Each macromolecule (e. g. e-RNA) is associated with a reaction responsible for producing it from its precursors (e. g. ATP, GTP, CTP, UTP).

The leaf macromolecular contents were based on data retrieved from *A*. *thaliana* models [15,32]. The cell wall sugar content was included in the e-Carbohydrate composition, while lignin was included in the e-Lignin composition. The monomer contents of DNA, RNA, and protein were determined using the biomass tool [33] available in *merlin*. The fatty acid, lipid, and carbohydrate compositions were determined using experimental data for *Q*. *suber* or closely related organisms when species-specific data was not available (Tables D, E, and H in S3 File) [34–36]. The lignin, carbohydrate, suberin, and wax contents and composition in the inner bark and phellogen were determined using available experimental data [37,38].

The Cofactors component includes a set of universal cofactors and vitamins [39]. These compounds were included in the biomass of each tissue. Nevertheless, the leaf “e-Cofactor” reaction also comprises a set of pigments, such as chlorophylls and carotenoids, determined according to experimental data [40].

The formulation of tissue-specific biomass composition is critical for obtaining tissue-specific models and predicting each tissue’s metabolic behaviour and the interactions among them. Thus, the information described above was compiled to formulate biomass general compositions for each tissue. The leaf is mainly composed of protein (43.7%) and carbohydrates (40.7%). Carbohydrates have a significant representation in the composition of the three tissues, being the major component of the inner bark (46.9%), together with lignin (38.20%). The phellogen is mainly composed of suberin (36.2%), with a considerable representation of carbohydrates (17.6%) and lignin (25.2%).

For tissue-specific model construction, reactions encoded by genes identified as “not expressed” were removed from each tissue-specific model. The number of genes and reactions decreased in rather different proportions, as *troppo* operates at the reaction level (See Material and Methods section 4.4). Table 4 shows the number of reactions and metabolites in the generic model and each tissue-specific model.

**Table 4:** General properties of the generic and tissue-specific GSM models. Genes were predicted based on the cork oak genome and then used to develop the GSM model. Orphan reactions: reactions without gene association (excluding exchange reactions). The reactions were divided according with the respective metabolic role: metabolic (enzymatic and spontaneous) and transport, and the respective compartment. The number of genes, metabolites, and reactions were determined in the generic model and each tissue-specific model, generated with *troppo*.

Component	Generic Model	Leaf	Inner Bark	Phellogen (reproduction)	Phellogen (virgin)
**Genes**	7871	7125	7004	7069	7044
**Metabolites**	6481	5222	5056	5239	5226
**Exchange reactions**	708	574	545	590	577
**Reactions (metabolic)**	6230	4514	4532	4587	4516
**Orphan Reactions**	353	344	344	345	345
** • Enzymatic/ Spontaneous**	4961	3528	3589	3572	3554
** • Transport**	1270	986	944	1017	962
** • Extracellular**	176	119	120	142	140
** • Plastid**	1355	984	939	947	934
** • Cytoplasmic**	1644	1151	1177	1171	1172
** • Mitochondria**	722	532	568	517	518
** • Peroxisome**	296	219	220	206	205
** • Vacuole**	130	87	76	93	90
** • Endoplasmic Reticulum**	583	391	443	453	451
** • Golgi Apparatus**	55	45	45	43	44

The number of metabolic reactions is quite similar among the four tissue-specific models. Nevertheless, the number of reactions in the leaf model’s plastid is slightly higher than in the other models, while in the endoplasmic reticulum, the opposite behaviour can be observed. The reproduction phellogen model presents a higher number of transport reactions than the remaining ones. To assess potential differences in the metabolism of the reproduction and virgin phellogen, *in silico* simulations with parsimonious flux balance analysis (pFBA) [41] and flux variability analysis (FVA) [42] were performed (Table H in S4 File).

The number of metabolic reactions associated with each pathway is available in Tables H-I in S2 File. The number of reactions associated with the “Arachidonic acid metabolism”, “Brassinosteroid biosynthesis”, and “Drug metabolism–cytochrome P450” pathways is higher in the inner bark and phellogen than in the leaf. The “Cutin, suberin and wax biosynthesis” pathway is mostly represented in the phellogen. As expected, the “Carotenoid biosynthesis” pathway is present in the leaf, while in the remaining tissues, the number of reactions is more limited. No significant differences were identified between the number of reactions in each pathway between the reproduction and virgin phellogen models.

### 2.4. Model validation

The models developed in this work were deposited in BioModels [43] and assigned the identifiers MODEL2205040001, MODEL2205040002, MODEL2205040003, MODEL2205040004, MODEL2205040005, and MODEL2205040006, together with the respective MEMOTE [44], and FROG (www.ebi.ac.uk/biomodels/curation/fbc) reports.

The models were tested to guarantee that no biomass or energy is produced without energy input in each condition. The general and tissue-specific metabolic models were validated by analysing the fluxes of *in silico* simulations in photoautotrophic, heterotrophic, and photorespiratory conditions. The exchange fluxes obtained in such simulations were not directly compared with measured rates due to the unavailability of this data. The results for the model’s validation are available in detail in S4 File and Tables A-F and Fig A-B in S5 File. All models grow in heterotrophic conditions, while in photoautotrophic and photorespiratory conditions only the general and leaf models produce biomass.

As expected, the photoautotrophic growth is supported by the assimilation of *CO*_2_ by RubisCO using light as the energy source through a C3 photosynthetic pathway. The predicted photon and *CO*_2_ uptake fluxes are 53.68 *mmol*∙*gDW*^−1^∙*h*^−1^ and 4.24 *mmol*∙*gDW*^−1^∙*h*^−1^, respectively, for a biomass production fixed at 0.11 *h*^−1^ (see Materials and Methods). These values are in the range of the ones reported by Collakova *et al*. (2012) [45] for AraGEM. In heterotrophic conditions using sucrose as the carbon source, the tricarboxylic cycle (TCA) and the oxidative phosphorylation become more active providing energy to sustain growth. The metabolic response to photorespiration was also assessed in the leaf model. In these conditions, the “Glyoxylate and dicarboxylate metabolism” pathway, which includes the reactions associated with photorespiration, becomes more active to recycle carbon skeletons (Fig A in S5 File).

Quantum yield (amount of *CO*_2_ fixed per mol of photons) and assimilation quotient (*CO*_2_ fixed per *O*_2_ evolved through photosynthesis) are common measures of photosynthetic efficiency. Thus, these parameters were determined for the leaf model. In photoautotrophic conditions, the photon yield was calculated as 0.079 ${mmol}_{{CO}_{2}}/{mmol}_{photon}$, which is within the range reported for *Q*. *suber* (0.051–0.089 ${mmol}_{{CO}_{2}}/{mmol}_{photon}$) at different light conditions and times of the year [2,46]. Quantum yield was also assessed in photorespiration by varying the ratio of carboxylation/oxygenation activity of Rubisco (reactions R00024 and R03140) using nitrate and ammonia as nitrogen sources (Fig B in S5 File). The results show that the model can predict a lower photosynthetic efficiency in drought conditions (carboxylation/oxygenation < 3.0), and when using nitrate as the nitrogen source. *In silico* simulations predict an assimilation quotient of 0.76 and 0.93 ${mmol}_{{CO}_{2}}/{mmol}_{O_{2}}$ with nitrate and ammonia as nitrogen source, respectively. This parameter reflects the quantity of *O*_2_ released by *CO*_2_ fixed to comply with the stoichiometry of biomass components [19]. These values are similar to the ones predicted in other plant leaf GSM models [19,47].

The inner bark and phellogen metabolic models produce all the metabolites defined in their biomass composition in heterotrophic conditions (Tables D-F in S5 File), using sucrose as the carbon source and obtaining energy through the TCA and oxidative phosphorylation. As expected, these models are not able to grow in photoautotrophic conditions as several genes associated with photosynthesis were considered as not expressed by *troppo*, thus, the respective reactions were removed.

### 2.5. Diel Multi-tissue model

**Fig 3:**
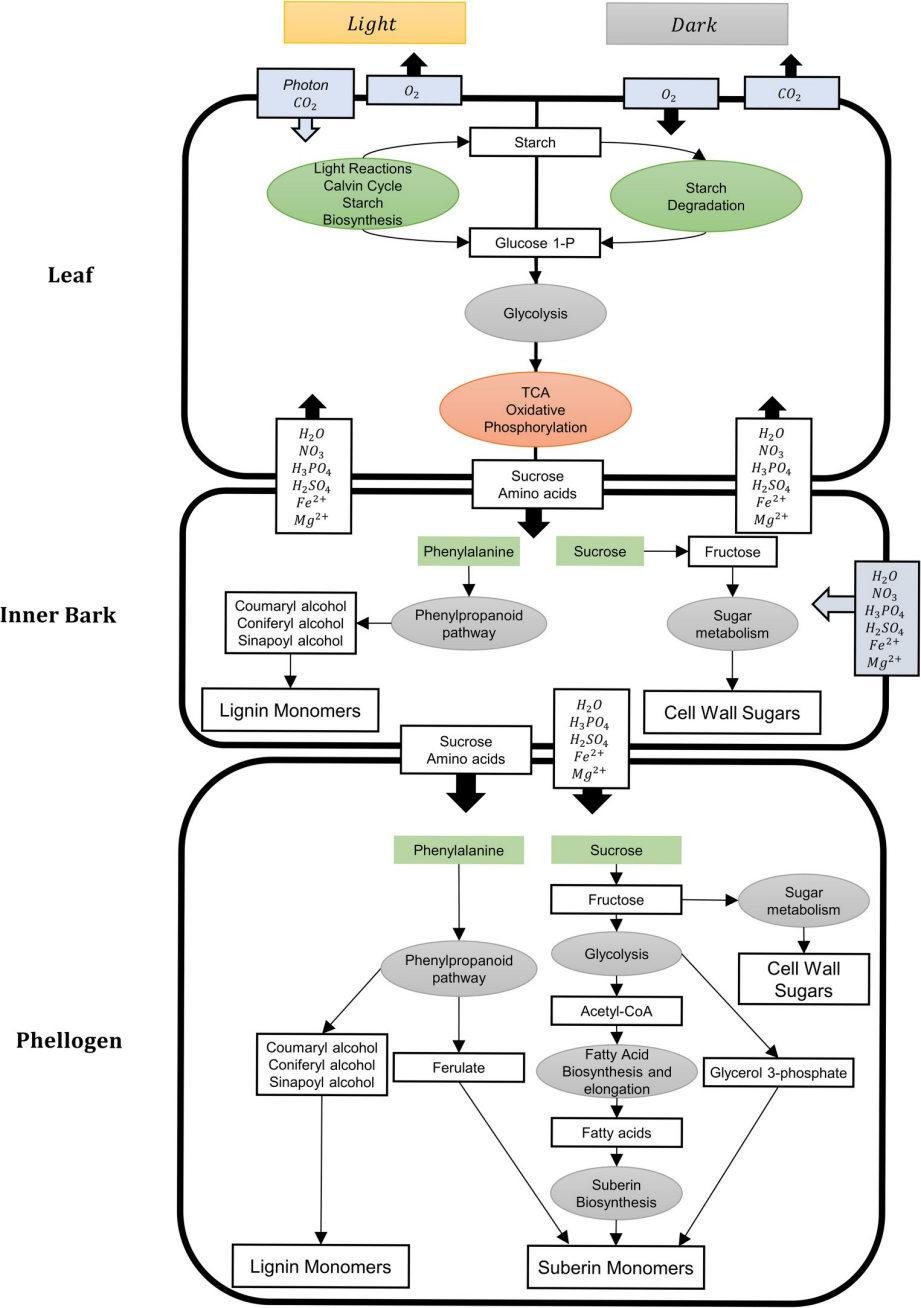
Schematic representation based on pFBA and FVA predictions of the metabolic routes towards cork formation. Photon and gas exchanges take place in the leaf, while the uptake of inorganic ions was assumed to happen in the inner bark. Sucrose and amino acids produced in the leaf are transported into the inner bark, and then to the phellogen, where they are used for the suberin and lignin biosynthesis (besides the other biomass components). The differences in the metabolism in the day and night phases are only represented for the leaf since, according to *in silico* simulations, the pathways used in the inner bark and phellogen were similar in the two phases of the diel cycle. Thick arrows represent transport reactions between tissues, while thinner arrows represent intracellular reactions.

A diel multi-tissue metabolic model was generated to analyse the metabolic interactions between leaf, inner bark, and phellogen at the two phases of the diel cycle: light (day) and dark (night). Common pools were created to connect the different tissues for the light and dark phases. The diel multi-tissue GSM model comprises 28,430 reactions and 29,489 metabolites. This model allows the uptake of minerals, such as *HNO*_3_ and *H*_3_*PO*_4_ through the inner bark since the root was not considered. The light/dark uptake ratio of nitrate was constrained to 3:2, as suggested in other diel GSM models [48,49]. The exchange of oxygen and carbon dioxide is only allowed in the leaves, where photons can be uptaken in the light phase. FVA and pFBA were computed, setting as the objective function the minimization of photon uptake for photorespiratory conditions. Fig 3 depicts both the relevant transport reactions between tissues, and the metabolites stored between the light and dark phases. The suberin, lignin, and cell wall sugars’ synthesis pathways are also portrayed. The detailed simulation results are available in Tables I-J in S4 File.

## 3. Discussion

### 3.1. Comparison with other models

*A*. *thaliana* and *Z*. *mays* are reference organisms for C3 and C4 plants, with several metabolic models available. Nevertheless, such models for less studied plants have been becoming available in the last decade [25,50–53]. The abundance of genetic and metabolic information for these organisms, in opposition to other plants like *Q*. *suber*, implies the utilization of this data to reconstruct metabolic models of less-studied organisms. Despite the manual curation efforts to assure network connectivity, especially for secondary metabolism pathways, the model still presents a high number of blocked reactions. This is associated with the lack of knowledge regarding enzyme specificity, and replication of reactions in multiple compartments.

The iEC7871 was compared with evidence-based *A*. *thaliana* and *Z*. *mays* models developed by Seaver *et al*. (2015) [18]. The iEC7871 presents 1,721 reactions and 1,436 metabolites not available in the other two models. Interestingly, the annotation of the genes associated with most of these reactions was based on *A*. *thaliana*’s homologs. The information available in Swiss-Prot for such species is exceptionally high, while it is scarce for woody plants. A taxonomic comparison between *A*. *thaliana* and *Q*. *suber* reveals a substantial phylogenetic divergence, as they belong to distinct orders: *A*. *thaliana* belongs to the order Brassicales, while *Q*. *suber* falls within the order Fagales. This divergence in evolutionary lineage may result in inaccurate or incomplete gene annotations, potentially leading to erroneous or missing information. Another obstacle is the presence of incomplete EC numbers. For instance, over 300 genes were initially annotated with incomplete EC numbers associated with Cytochromes P450.

The KEGG Pathways associated with these reactions were also analysed. The pathways with a higher number of unique reactions are associated with the metabolism of fatty acids, xenobiotics, terpenoids, and other secondary metabolites. Also, the “Tryptophan metabolism” and “Tyrosine metabolism” pathways include the secondary metabolism of these amino acids, such as the quinolinic acid production, dopamine, and its derivates.

When classified according to the main areas of the metabolism in KEGG, the pathways representing “Lipid metabolism”, “aminoacid metabolism and carbohydrate metabolism”, and “Xenobiotics biodegradation metabolism” were the most represented regarding reactions that are only present in iEC7871.

The high number of reactions and metabolites only available in the iEC7871 is most likely associated with three different reasons: the approach followed to develop the GSM model; the different databases used for the reconstruction of the models, which has been pointed to have a major impact on the reconstruction of metabolic models [54]; the metabolic differences between the three species.

Seaver *et al*. (2015) [18] used both KEGG and PlantCyc to reconstruct full *A*. *thaliana* and maize models, leading to a remarkably high number of metabolites and reactions. These models were screened by the authors, and only genes for which evidence in PlantSEED or PlantCyc was found were kept in the evidence-based models. Thus, these models are mostly based on PlantCyc identifiers, which increases the number of mismatches between the models, as mapping identifiers between this database and KEGG is not straightforward.

### 3.2. Secondary metabolism pathways

Plants produce a wide range of compounds through their secondary metabolism, whose function includes defence against abiotic and biotic stress or beneficial interactions with other organisms [55]. Many secondary metabolites have central roles in the pharmaceutical, cosmetics, perfume, dye, and flavour industries. Despite the recent advances in the investigation of plant secondary metabolism, detailed knowledge of these pathways is restricted to a few species [55], such as *A*. *thaliana*, *O*. *sativa*, and *Z*. *mays*. These pathways are not always complete or available in biological databases, implying an additional effort to find information regarding genes, reactions, and metabolites. Although there is still much to learn about these compounds and their respective biosynthetic pathways, genome-scale modelling can provide insights into a given organism’s potential to produce secondary metabolites. The model reconstructed in this work includes reactions associated with the biosynthesis and metabolism of several secondary metabolites, steroids, and drugs.

The “Metabolism of xenobiotics by cytochrome P450” includes 56 reactions not found in the *A*. *thaliana* and *Z*. *mays* models considered in the Results section. This pathway represents a set of reactions, mostly associated with the cytochrome P450, responsible for the response to the presence of toxic xenobiotics. The cytochrome P450 family has been described as being highly upregulated in developing phellem tissues [31]. Nevertheless, members of this protein family are highly promiscuous [56–59], and probably involved in the ω-hydroxylation of fatty acids for suberin monomers biosynthesis in *Q*. *suber* [31,60]. Further analysis of the annotation of these genes would be useful to better characterize their metabolic function in the cork oak tree, as homology-based annotation is not able to determine the substrate specificity of Cytochrome P450 enzymes.

The model presents all the necessary reactions to produce jasmonic acid and its derivates, usually called jasmonates, through the “alpha-Linoleic acid metabolism” pathway. These plant hormones are associated with the regulation of growth and developmental processes, stomatal opening, inhibition of Rubisco biosynthesis, and nitrogen and phosphorus uptake [61,62]. The “Steroid biosynthesis” pathway is essentially complete, enabling the production of steroids like ergosterol, cholesterol, and stigmasterol. The latter is known as one of the most abundant plant sterol components, while cholesterol has been found as a minor component [63,64]. The enzymes associated with the synthesis of calcitetrol and secalciferol (animal hormones) are the only ones whose encoding genes were not found in the genome of *Q*. *suber*. Although these hormones are usually synthesized by animals, they were already identified in a few plants [65–67]. The biosynthetic pathway for the synthesis of gibberellins is also available in the model. Although the regulatory effect of hormones and steroids cannot be quantitively evaluated in stoichiometric models, the presence of their biosynthetic pathways, connected to the core network, can be used as a source of information regarding the potential to produce a certain hormone or steroid.

Although this model contains a considerable amount of information regarding secondary metabolism, further curation using species-specific knowledge would improve the connectivity of the model and reduce the existing gaps in these pathways.

### 3.3. Suberin, Lignin, and Waxes Biosynthesis

**Fig 4:**
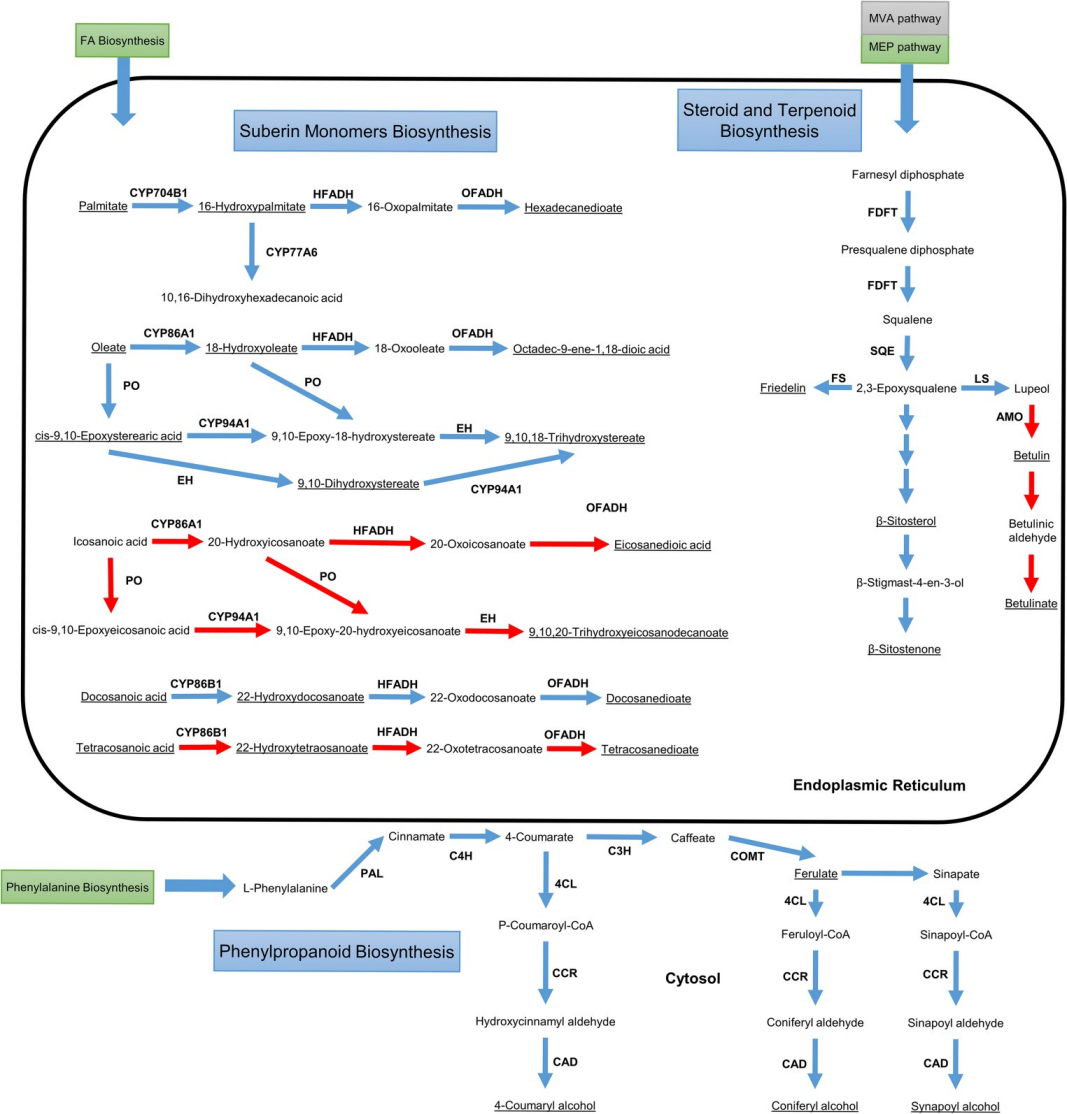
Biosynthetic pathway of suberin, waxes, and lignin monomers. Reactions not available at KEGG are highlighted in red. The components of suberin, lignin, and waxes are underlined. The C:16 and C:18 fatty acids produced in the plastid are transported to the endoplasmic reticulum, where they can be elongated and unsaturated, and follow the suberin monomer biosynthesis pathway. Cytochromes P450 (CYPs), peroxygenases, epoxy hydroxylases, ω-oxo-fatty acid dehydrogenases, and ω-hydroxy-fatty acid dehydrogenases catalyse successive reactions to produce the aliphatic monomers. Farnesyl diphosphate, produced from isopentenyl diphosphate provided by the cytosolic mevalonate (MVA) pathway or by the plastidic methylerythritol phosphate (EMP) pathway is used as the initial precursor of steroids in the ER. 2,3-Epoxysqualene is converted into sterols and terpenoids, monomers of the wax component of the phellogen. The phenylpropanoid pathway uses phenylalanine produced in the leaf’s chloroplasts, to produce cinnamate. The pathway follows in the cytosolic surface of the endoplasmic reticulum, and then in the cytosol, producing the lignin monomers. 4CL– 4-Coumarate CoA ligase, AMO: β-amyrin monooxygenase, C3H: p-coumarate 3-hydroxylase, C4H: cinnamate 4-hydroxylase, CAD: cinnamyl alcohol dehydrogenase, CCR: cinnamoyl-CoA reductase, COMT: Caffeoyl-CoA O-methyltransferase, EH: epoxide hydroxylase, FDFT: farnesyl diphosphate farnesyltransferase, FS: friedelin synthase, HFADH: ω-hydroxy-fatty acid dehydrogenase, LS—lupeol synthase, OFADH: ω-oxo-fatty acid dehydrogenase, PAL: phenylalanine ammonia-lyase, PO: peroxygenase, SQE: squalene monooxygenase. *A*. *thaliana* identifiers were used for the nomenclature of CYPs for convenience. The IDs of the reactions catalysed by these enzymes can be found in Table L in S2 File.

As mentioned before, suberin is the major component of phellogen, while its abundance is residual in the leaf and inner bark. The phellogen also contains significant amounts of lignin and waxes. The biosynthesis pathway of the monomers of these cork components in the model is represented in Fig 4.

The synthesis of aliphatic suberin monomers is associated with the KEGG’s “Cutin, suberine, and wax biosynthesis” pathway. Nevertheless, the reactions of this pathway in the database are associated only with palmitic, oleic, and docosanoic acids and some of their derivates. Hence, additional reactions and metabolites were added to allow the production of the components of suberin, and waxes identified in the biomass formulation stage.

Fatty acids are exported from the plastid and imported into the endoplasmic reticulum, where these can be elongated to originate long-chain and very-long-chain fatty acids. Cytochromes P450, especially homologues of the CYP86A family, are responsible for catalysing several reactions that synthesize suberin monomers. The proteins with NCBI accessions XP_023927575 (CYP86A1) and XP_023893592, XP_023920300, XP_023893496, XP_023896997, XP_023896998, and XP_023871406 (CYP704B1) are responsible for the ω-hydroxylation of long-chain fatty acids, while XP_023897881, XP_023929457, XP_023923098, and XP_023874080 (CYP86B1) act on very-long-chain fatty acids.

Epoxide hydrolases (EC: 3.3.2.10), ω-oxo-fatty acid dehydrogenases (EC: 1.2.1.-), ω-hydroxy-fatty acid dehydrogenases (EC: 1.1.1.-), and peroxygenases (EC: 1.11.2.3) can act in the ω-hydroxy acid in different combinations, to originate the diverse α,ω-dicarboxylic, poly α,ω-dicarboxylic, and polyhydroxy-fatty acids. The genome annotation and the model manual curation allowed identifying 38 genes encoding epoxide hydrolase, five encoding ω-hydroxy-fatty acid dehydrogenase, and six encoding peroxygenases. Nevertheless, no genes encoding ω-oxo-fatty acid dehydrogenase were available in known biological databases; thus, the reactions catalysed by this enzyme (“R09458” and “R09459”) were added without gene association.

The polymerization process is still not completely understood but it likely involves esterification reactions through glycerol-3-phosphate acyltransferases (GPAT), producing acylglycerol esters [68,69]. This family of enzymes was identified as a key step in suberin/cork polymerization. The expression level of GPAT5 gene is higher in June, which corresponds to a period of higher phellogen activity [70]. Acylglycerol esters are then secreted through the Golgi secretory pathway and ABC transporters (which are overexpressed in phellem [31]) and incorporated into the suberin glycerol polyester [71]. The aliphatic suberin polyester transport and assembly were simplified in the model and represented through a reaction converting the determined precursors (Table F in S3 File) into the macromolecular representation of suberin: “e-Suberin”.

One of the major differences between virgin and reproduction cork is the suberin content [38]. Thus, the FSEOF method was applied to identify genes and reactions that promote suberin production. As expected, reactions associated with the suberin monomers’ biosynthesis pathways were identified here. Glycerol-3-phosphate is produced in a reaction catalysed by glycerol-3-phosphate dehydrogenase, while ferulate is formed through the phenylpropanoid biosynthesis pathway. Regarding fatty acid metabolism, the reaction R09452 was identified as highly influencing suberin production. This reaction is responsible for converting oleate into 18-hydroxyoleate (Fig 4). In addition to constituting a substantial portion of the suberin composition (Table F in S3 File), this compound is also a precursor of other components like octadic-9-ene-1,18-dioic acid and 9,10,18-trihydroxystereate. As mentioned above, cytochrome P450 enzymes are involved in several reactions associated with this pathway. The reductive power of these enzymes is restored using NADPH by cytochrome P-450 reductase, which was also identified using the evaluation function.

Waxes are also an important cork component and are composed essentially of sterols and terpenoids [72]. These are produced through the “Steroid biosynthesis” pathway in the endoplasmic reticulum. The initial precursor, farnesyl diphosphate, is synthesized in the cytosolic mevalonate pathway or by the plastidic methylerythritol phosphate pathway. Farnesyl diphosphate farnesyltransferase and squalene monooxygenase convert farnesyl diphosphate into 2,3 –epoxysqualene. This triterpenoid can be converted into cyclic triterpenoids, such as friedelin and lupeol, or can be used to produce phytosterols like β-sitosterol, through a longer pathway.

The phellogen contains a considerable amount of lignin, although this polymer is present in other tissues. The synthesis of the respective monomers (guaiacyl lignin, hydroxyphenyl lignin, and syringyl lignin) is described in the KEGG’s “Phenylpropanoid biosynthesis” pathway. Phenylalanine is the main precursor of this pathway that occurs mostly in the cytoplasm. It is converted to cinnamic acid by phenylalanine ammonia-ligase (EC: 4.3.1.24). Cinnamate monooxygenase, caffeate methyltransferase, and ferulate-5-hydroxylase are responsible for the successive conversion of cinnamic acid to coumarate, ferulate, hydroxyferulate, and sinapic acid, in the cytosolic surface of the endoplasmic reticulum. These metabolites can be converted into lignin monomers in a three-step process catalysed by coumarate-CoA ligase (EC: 6.2.1.12), cinnamoyl-CoA reductase (EC: 1.2.1.44), and cinnamyl-alcohol dehydrogenase (EC: 1.1.1.195). Through the polymer assembly in the cell wall, peroxidases (EC: 1.11.1.7) convert the guaiacyl, hydroxyphenyl, and syringyl alcohols into guaiacyl, hydroxyphenyl, and syringyl lignins, respectively. The linkages between aliphatic and aromatic suberin were not included in the model since these macromolecules are represented in the biomass separately.

These findings pave the way for further research and engineering approaches to enhance suberin production. By overexpressing the identified target reactions or manipulating the associated metabolic pathways, it becomes feasible to fine-tune the biosynthesis of suberin and potentially achieve higher yields. This has implications not only for cork quality and properties but also for various applications that rely on suberin, such as sustainable packaging, biodegradable materials, and agricultural protection. The identified reactions serve as valuable starting points for experimental investigations and provide a foundation for understanding the underlying metabolic mechanisms involved in suberin production.

### 3.4. Tissue-specific models

A generic metabolic model comprises all the reactions catalysed by all the enzymes encoded in an organism’s genome. However, this approach does not account for the regulatory network present in each organ, tissue, or cell in different environmental conditions. The integration of transcriptomics data in a GSM model allows obtaining metabolic models closer to the *in vivo* phenotype of the respective tissue or condition [73].

Transcriptomics data for the leaf, inner bark, and phellogen were integrated into the generic model, originating three tissue-specific models with different biomass compositions. The leaf macromolecular composition was mostly based on previously published GSM models [15,32,74]. Quantitative information regarding the leaf composition of *Q*. *suber* would be useful to increase the model’s reliability and improve predictions. The composition of the inner bark and phellogen was based on experimental data available for *Q*. *suber* [37,75]. While the inner bark is mostly composed of lignin and carbohydrates, the phellogen also comprises suberin and waxes. Suberin is composed of glycerol, ferulic acid, and diverse alkanols, fatty acids, hydroxy acids, ω-hydroxy acids and α,ω-dicarboxylic acids [76]. Waxes are composed of terpenes, such as friedelin, and sterols, like β-sitosterol. Although the cork bark contains tannins (mostly ellagitannins) [77], these compounds were not accounted for since their biosynthetic pathways are poorly understood and are not available in the used biological databases.

The number of transport reactions in the Reproduction Phellogen is visibly higher than in the other tissues, including the Virgin Phellogen. These reactions are essentially associated with the transport of steroids and hormones. Based on BLAST searches against TCDB [78], the genes associated with these reactions are auxin (phytohormone required for cell division [79]) efflux pumps. An analysis of the pathways available in the Reproduction and Virgin Phellogen models, followed by *in silico* simulations did not allow identifying significant differences between them. The cork oak phellogen’s transcriptional profile allows inferring that the flavonoid route is favoured in bad quality cork, while the lignin and suberin production pathways are preferred in good quality cork production [80]. A recent study reported a higher expression of genes associated with fatty acid biosynthesis and elongation in reproduction cork [31]. Although it was reported that the macromolecular composition of reproduction and virgin cork could be similar, the first usually presents higher amounts of suberin and a lower quantity of extractives [38,76]. Hence, a biomass composition specific to the virgin phellogen should be defined, including the tannin content, to allow the observation of metabolic differences between the two models.

### 3.5. Diel multi-tissue model

Tissues and organs of multicellular organisms do not perform their metabolic functions individually. Instead, they interact with each other by exchanging sugars, amino acids, hormones, and others. In plants, the simplest example is the sucrose formation in the leaves and its transport across all other organism tissues. Multi-tissue GSM models allow analysing the dependencies between tissues in terms of biomass precursors, carbon skeletons, nitrogen, sulphur and phosphorus sources, and the energy required for their translocation and proton balance [12,81]. The introduction of light and dark phases allows predicting features that are not possible in continuous light models, such as the accumulation and utilization of carboxylic acids [48].

The leaf, inner bark, and phellogen models were merged into a multi-tissue GSM model. Other tissues, such as the root and xylem, could also be considered and included in the multi-tissue model. Nevertheless, this work’s scope is related to the metabolism of cork precursors and including more tissues would increase the complexity and computational requirements of the model unnecessarily.

The most significant differences between the light and dark phases of metabolism were identified in the leaf (Tables I-J in S4 File), due to the photosynthesis and photorespiration pathways.

In the light-dependent reactions, carbon dioxide is fixed through the Calvin Cycle, forming carbohydrates, and consequently, the carbon skeletons for the synthesis of the remaining biomass components. A fraction of the carbohydrates produced here (mainly starch) is stored and used as energy source when needed. The storage of starch is more efficient in the model than sucrose, glucose, or fructose. Whereas starch is mobilized in plastids, soluble sugars are accumulated in the vacuole, implying an energetic cost for their transport. The model produces malate in the dark (light-independent reactions), which is used during the day (light-dependent) by the malic enzyme to produce pyruvate and obtain reducing power (NADPH). Citrate was also produced at night (light-independent reaction) through the tricarboxylic cycle (TCA) and used to feed the diurnal TCA and provide carbon skeletons for the amino acid metabolism. This behaviour is in agreement with the previously published diel GSM model for *A*. *thaliana* [48].

For the inner bark and phellogen, the metabolic profile was very similar between day and night. This is associated with the metabolic constraints applied to the model: for both phases, the leaf produces and transports sucrose and amino acids to the inner bark, and then to the phellogen. Thus, as the nutrient supply is very similar, and the objective function is the same (biomass production), the simulations applied here are not able to distinguish daytime and nighttime metabolism. Nitrate provided by the inner bark is transported into the leaf and is then converted into ammonia. In the plastids, ammonia is used to incorporate nitrogen through the glutamine synthetase-glutamate synthase pathway.

Sucrose and amino acids are exported from the leaf and then imported by proton symport by the inner bark. The proton balance of the common pool is maintained by plasma membrane ATPases. In the inner bark, sucrose is used as an energy and carbon source to produce biomass components. As mentioned before, the inner bark biomass is mainly composed of carbohydrates, lignin, and protein. Hence, pathways associated with the synthesis of cell wall precursors (“Amino sugar and amino nucleotide metabolism”, “Starch and sucrose metabolism”, and “Phenylpropanoid biosynthesis”) are the most relevant in this tissue.

The remaining sucrose and amino acids produced in the leaves are transported to the phellogen. The reversible sucrose synthase activity allows sucrose conversion into UDP-glucose, which is used to produce other cell wall components, and fructose that follows the glycolytic pathway to produce pyruvate. A fraction of it is transported into the plastid and converted by pyruvate dehydrogenase into acetyl-CoA, the fatty acids precursor. As mentioned above, fatty acids are elongated and suffer a series of hydroxylation, epoxidation, and peroxidation reactions in the endoplasmic reticulum. Ferulate, another precursor of suberin, is produced through the “Phenylpropanoid biosynthesis” pathway, with phenylalanine produced in the leaf serving as a precursor. The glycerol 3-phosphate used for the esterification reactions is produced from glycerone phosphate produced through glycolysis.The diel multi-tissue GSM model developed in this work is a useful framework capable of providing a systematic overview of the metabolism of *Q*. *suber* at a global level. It can be used to study the biosynthetic pathways of suberin, lignin, waxes, and several plant secondary compounds. The introduction of additional species-specific knowledge would improve the overall network connectivity, allowing obtaining a model with increased reliability.

## 4. Conclusions

In this work, we present the first multi-tissue diel genome-scale metabolic model of a woody plant. The iEC7871 was based on knowledge retrieved from genomic information, biological databases, and literature. It can simulate the Cork Oak tree’s metabolic behaviour in phototrophic and heterotrophic conditions, as well as photorespiration. This model comprises the pathways of the central metabolism and several pathways associated with the secondary metabolism reproducing the formation of the major components of cork.

The integration of transcriptomics data was used to obtain tissue-specific models for the leaf, inner bark, and phellogen. These models were merged to obtain a multi-tissue GSM model that comprises the diel cycle’s dark and light phases.

This GSM model comprehends the four main secondary metabolic pathways participating in cork production: acyl-lipids, phenylpropanoids, isoprenoids, and flavonoids. The lipid biosynthesis pathway is required for the biosynthesis of the linear long-chain compounds forming the aliphatic suberin domain, which share upstream reactions with wax biosynthesis. The phenylpropanoid metabolism is needed for the biosynthesis of the cork aromatic components, which share reactions with wood lignin.

The metabolic models developed in this work can be used as a tool to analyse and predict the metabolic behaviour of the tree and evaluate its metabolic potential. Metabolic modelling methods can be applied, including dynamic approaches, to study the changes in this tree’s metabolism over time and environment.

## 5. Materials and methods

### 5.1. Software

*merlin* v4 was used to support the reconstruction process, as this software provides a user-friendly interface that conjugates automatic and manual procedures. COBRApy v0.20.0 [82] was used to perform all simulations and analyses of the GSM model, as well as generate the diel multi-tissue model. The simulations were performed using the CPLEX v128.0.0 solver.

The *troppo* [30] python package, compliant with COBRApy models, was used to integrate the transcriptomics data in the metabolic model, originating tissue-specific models.

FastQC (https://www.bioinformatics.babraham.ac.uk/projects/fastqc/), Sickle [83], Bowtie2 [84], FeatureCounts [85], and edgeR [86] were used to process the transcriptomics data.

### 5.2. Metabolic reconstruction

The “Automatic workflow” tool, available in *merlin*, was used to perform the genome annotation by assigning EC numbers to enzyme-encoding genes based on BLAST searches against Swiss-Prot and TrEMBL, using an e-value threshold of 1e^-30^. This workflow allows annotating genes based on a pre-defined set of well-studied phylogenetically close related species (Table A in S1 File).

A draft metabolic network was assembled by loading KEGG’s metabolic information and integrating the genome annotation. KEGG reactions associated with enzymes identified in the genome annotation stage were included in the model by *merlin*, as well as spontaneous reactions.

Transport reactions were automatically generated using the TranSyT [87] tool directly in *merlin* using default parameters. This tool annotates transport genes using the Transporter Classification system based on homology searches against TCDB. Then, the transport reactions are generated based on the annotation using information available at TCDB, KEGG, and MetaCyc. Nevertheless, additional transport reactions were added if reported in the literature, or if necessary for the model functionality. The subcellular location of proteins was predicted using the WoLF PSORT [88] tool. Additionally, LocTree3 [89] and ChloroP 1.1 [90] were used to verify protein location predictions during the manual curation stage. The compartment annotation for all proteins obtained using WoLF PSORT and LocTree3 is available in Table O in S2 File.

The leaf, inner bark, and phellogen biomass compositions were based on previously published plant GSM models and available literature. The biomass precursors were organized in macromolecules or cell structures, labelled as “e-Metabolites”. The leaf macromolecular contents were determined using *A*. *thaliana* models [15,32]. The cell wall sugar content was included in the e-Carbohydrate composition, while lignin was included in the e-Lignin composition. The monomer contents of DNA, RNA, and protein were determined using the biomass tool, available in *merlin*. The fatty acid, lipid, and carbohydrate compositions were determined using experimental data for *Q*. *suber* or closely related organisms when species-specific data were not available [34–36]. The lignin, carbohydrate, suberin, and wax contents and composition in the inner bark and phellogen were determined using available experimental data [37,38]. Suberin was only accounted for in the inner bark and phellogen. The e-Cofactor component includes a set of universal cofactors and vitamins [39], which were included in the biomass of each tissue. Nevertheless, the leaf “e-Cofactor” reaction also comprises a set of pigments, such as chlorophylls and carotenoids, determined according to experimental data [40]. The energy requirements were inferred as reported by [91].

Through the manual curation process, literature and biological databases, namely KEGG, MetaCyc [92], and BRENDA [93], were consulted to retrieve information regarding metabolic, genomic, and enzymatic information, as described previously [94,95]. Briefly, KEGG Pathways allowed identifying reactions that were not included through the automatic network assembly. The inclusion of such reactions was evaluated after reviewing the genome annotation to identify potential gene candidates to encode the enzyme associated with the reaction. MetaCyc was used to identify additional pathways/reactions not available in KEGG; MetaCyc and BRENDA provided information regarding the reaction’s reversibility and mass-balance. Since KEGG reactions often contain generic representations of carbohydrates (e.g., D-glucose, instead of alpha-D-glucose or beta-D-glucose), the isomer usage by enzymes was determined using information retrieved from BRENDA. COBRApy and *merlin* functionalities were used to identify gaps in the network, which were corrected when information was found in the above-mentioned databases and/or literature.

The validation of the GSM model started by assuring the biomass formation, which was performed using the BioISO plug-in available in *merlin*.

The following validation approaches were applied to the model using FBA, pFBA and FVA, available in COBRApy:

1. Growth without photons, carbon, nitrogen, phosphorus, and sulfur sources.
2. Futile cycles and stoichiometrically balanced cycles.
3. Growth rate assessment
4. Growth with different elemental sources.
5. Capacity to present flux through photosynthesis, respiration, and photorespiration.

### 5.3. Comparison with other models

The evidence-based models developed by Seaver *et al*. (2015) [18] were compared with the iEC7871. First, the compartments were removed from the identifiers of reactions and metabolites. Thus, if the same reaction was available in more than one compartment, it was only accounted for once. Then, these identifiers were converted into MetaNetX IDs, using the cross-references available at this database. The identifiers of the Chung *et al*. (2013) [24] and Saha *et al*. (2011) models were directly compared after removing the compartments since they present KEGG IDs.

### 5.4. Tissue-Specific and multi-tissue models

Transcriptomics data from different tissues were used to obtain tissue-specific models. The quality of the transcriptomics data was analysed using the FastQC software. After trimming the data with Sickle, Bowtie2 was used to align the trimmed FASTQ files against the reference genome. FeatureCounts was used to count the mapped reads to each gene. After filtering and normalizing the data with the edgeR library, datasets with the normalized counts of each gene were obtained. These files were used as input for *troppo*, together with the generic GSM model, to generate tissue-specific models. *Troppo* was executed using the CPLEX solver and the Fastcore [96] reconstruction algorithm. A *troppo*’s integration strategy was developed to guarantee that the tissue-specific models can produce biomass. The median of each dataset was used as threshold, and the remaining parameters were kept as default. *Troppo* identifies the reactions that should be removed from the generic model, based on the expression of the genes included in the gene-protein-reaction association of each reaction. Nevertheless, the reactions that were maintained in the model within this approach kept all their respective genes. The carotenoid biosynthesis has two branches, one associated with trans-Phytofluene and the other with 15,9-dicis-Phytofluene. The enzymes present in the two branches are similar (phytoene desaturase and zeta-carotene desaturase). However, one of them is dependent on photosynthesis since it unbalances the plastoquinone/plastoquinol ratio. The other one is stoichiometrically balanced since it involves successive oxidation and reduction of these quinones. Hence, the presence of the second branch was guaranteed in the leaf model since it is essential for carotenoid production through the dark phase.

The tissue-specific models obtained using *troppo* were adapted to account for the light and dark conditions, by duplicating all reactions and metabolites. Reactions converting sugars (starch, sucrose, glucose, fructose, malate, citrate, quercitol, and quinate), 18 amino acids, and nitrate between light and dark were added to the model, based on a previously published approach [48].

To build the multi-tissue model, the diel tissue-specific models were merged, and common pools between leaf and inner bark (common pool 1), and inner bark and phellogen (common pool 2) were created. Since the root was not accounted for, the uptake of water, nitrogen, sulphur, phosphorus, and magnesium sources was included in the inner bark.

### 5.5. Simulations

Two different approaches were used to perform simulations with pFBA. Since no experimental data regarding exchange fluxes for *Q*. *suber* was found, simulations were carried out with arbitrary values, and only flux ratios were evaluated. The first approach was to fix the biomass formation to 0.11 *h*^−1^, and set the minimization of the photon uptake as the objective function. In the second approach, the photon uptake was fixed to 100 *mmol*_*photon*_. *gDW*^−1^. *h*^−1^, maximizing biomass production. Photorespiration was simulated by constraining the carboxylation/oxygenation flux ratio (Vc/Vo) of Rubisco (reactions R00024 and R03140) to 3:1 [97]. Simulations with the inner bark, and virgin and reproduction phellogen were performed by maximizing the biomass production while limiting the sucrose and amino acid uptake to 1 *mmol*_*photon*_. *gDW*^−1^. *h*^−1^.

The pFBA and FVA simulations with the diel multi-tissue GSM model were performed using the restrictions applied in photorespiration, with the additional constraint of the nitrate light/dark uptake ratio, which was settled to 3:2 [48,49].

### 5.6. Flux scanning based on enforced objective flux

To explore differences between virgin and reproduction cork through suberin production, the FSEOF approach was applied to the generic metabolic model [28]. First, suberin was removed from the biomass reaction. Then, the production of suberin (*v*_*suberin*_) was maximized to obtain the theoretical maximum production of this compound. The production of suberin was enforced from 5% to 95% of the value calculated previously, using the growth rate (*v*_*biomass*_) as the objective function for FBA simulations. The reaction’s fluxes were analysed, to identify those that increased with higher suberin production rates. The identified targets were evaluated using FVA, and only those that satisfy $v_{j}^{max}∙v_{j}^{min}>0$ were considered. Finally, overexpression (OE) was simulated by enforcing the flux of each one of these reactions to 1.2 of the wild-type (WT) flux. The phenotypic fraction, *f*_*ph*_, was used to rank the identified targets, and was calculated as:

$$f_{ph}=\frac{v_{suberin,OE}}{v_{suberin,WT}}∙\frac{v_{biomass,OE}}{v_{biomass,WT}}$$

### 5.7. Accession numbers

The genome sequence of *Quercus suber* was retrieved from the NCBI database with the assembly accession number GCF_002906115.1. The transcriptomics data was retrieved from the EBI database [98] (Accession PRJNA392919 for Leaf and Inner Bark; Accession PRJEB33874 for Phellogen).

## Supporting information

S1 File Results of the genome annotation, and respective analysis. (XLSX)

S2 File Analysis of reactions and metabolites included in the iEC7871 and metabolic models of *Arabidopsis thaliana* and *Zea mays*. (XLSX)

S3 File Biomass composition. Data collected from publications, and respective processing to include it in the GSM models.
(XLSX)

S4 File Flux values for simulations obtained with pFBA and FVA under different environmental conditions using the different models. (XLSX)

S5 File Metabolic map of carbon and nitrogen assimilation through photorespiration (Fig A). Analysis of the quantum yield for different carboxylation/oxygenation ratio of Rubisco (Fig B). Summary of the simulations detailed in S4 File.
(PDF)
