# Supplementary material for: The first multi-tissue genome-scale metabolic model of a woody plant highlights suberin biosynthesis pathways in Quercus suber
Source: PLoS Comput Biol. 2023 Sep 20;19(9):e1011499. doi: 10.1371/journal.pcbi.1011499 (PMC10545120; doi:10.1371/journal.pcbi.1011499)
Supplement: S5 File — Analysis of the quantum yield for different carboxylation/oxygenation ratio of Rubisco (Fig B). Summary of the simulations detailed in S4 File. (PDF) [file pcbi.1011499.s005.pdf]

The first multi-tissue genome-scale metabolic model of a woody plant highlights suberin biosynthesis pathways in *Quercus suber*

Emanuel Cunha[1,2], Miguel Silva[1,2], Ines Chaves[3;4], Huseyin Demirci[1,2,5], Davide Rafael Lagoa[1,2], Diogo Lima[1,2], Miguel Rocha[1,2], Isabel Rocha[3, ¶,*], and Oscar Dias[1,2, ¶,*]

[1] Centre of Biological Engineering, Universidade do Minho, 4710-057 Braga, Portugal

[2] LABBELS – Associate Laboratory, Braga, Guimarães, Portugal

[3] Instituto de Tecnologia Química e Biológica António Xavier, Universidade Nova de Lisboa, Avenida da República, Quinta do Marquês, 2780-157 Oeiras, Portugal

[4] iBET, Instituto de Biologia Experimental e Tecnológica, Apartado 12, 2781-901 Oeiras, Portugal

[5] SnT/University of Luxembourg, Luxembourg

¶ Senior authors

* Corresponding authors: irocha@itqb.unl.pt; odias@ceb.uminho.pt

Fig. A Metabolic map of carbon and nitrogen assimilation, and photorespiratory pathway during photorespiration based on the simulation mentioned in Table S3.

Fig. B Variation of the quantum yield (mol CO_2 fixed per mol of photon) with the carboxylation/oxygenation ratio (Vc/Vo) of Rubisco.

Table A Summary of a pFBA applied to the leaf model in photoautotrophic conditions.

Table B Summary of a pFBA applied to the leaf model in heterotrophic conditions.

Table C Summary of a pFBA applied to the leaf model in photorespiratory conditions.

Table D Summary of a pFBA applied to the inner bark in heterotrophic conditions

Table E Summary of a pFBA applied to the virgin phellogen model in heterotrophic conditions.

Table F Summary of a pFBA applied to the reproduction phellogen model in heterotrophic conditions.

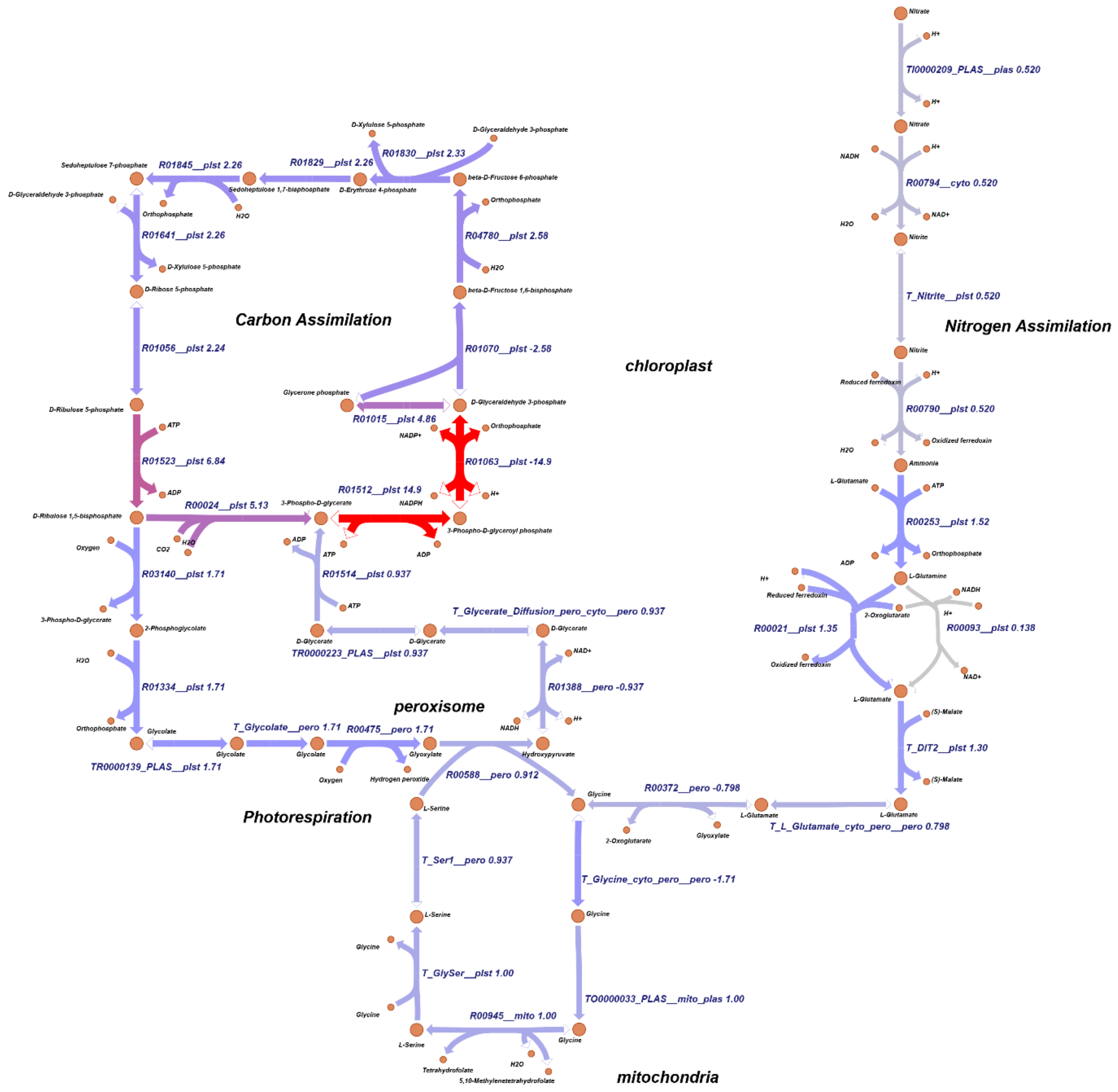

Fig. A Metabolic map of carbon and nitrogen assimilation, and photorespiratory pathway during photorespiration based on the simulation mentioned in Table S3.

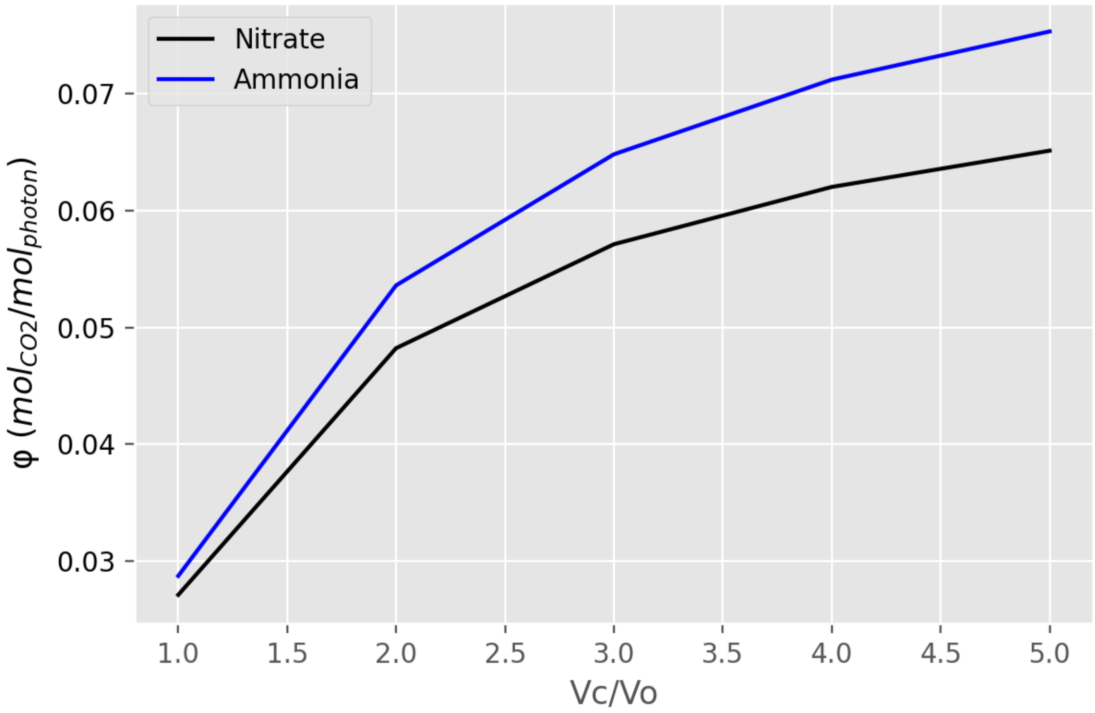

Fig. B Variation of the quantum yield (mol CO2 fixed per mol of photon) with the carboxylation/oxygenation ratio (Vc/Vo) of Rubisco. The biomass was fixed to 0.11 /h and the objective function was defined as the minimization of photon uptake.

Table A Summary of a pFBA applied to the leaf model in photoautotrophic conditions. The Biomass was fixed to 0.11 /h and the objective function was defined as the minimization of photon uptake.

REACTION	METABOLITE	FLUX
**EX_C00205__DRA**	hn	-53.674812
**EX_C00011__DRA**	CO_2	-4.236668
**EX_C00001__DRA**	H_2O	-2.817708
**EX_C00244__DRA**	Nitrate	-0.520394
**EX_C00059__DRA**	Sulfate	-0.016141
**EX_C00009__DRA**	Orthophosphate	-0.007504
**EX_C00080__DRA**	H^+	-5e-06
**EX_C00305__DRA**	Magnesium cation	-2e-06
**EX_C14818__DRA**	$Fe2^+$	-1e-06
**EX_C00237__DRA**	CO	2e-06
**EX_BIOMASS__CYTO**	e-Biomass	0.11
**EX_C00007__DRA**	Oxygen	5.582437

Table B Summary of a pFBA applied to the leaf model in heterotrophic conditions. The Biomass was fixed to 0.11 /h and the objective function was defined as the minimization of sucrose uptake.

REACTION	METABOLITE	FLUX
**EX_C00007__DRA**	Oxygen	-1.810577
**EX_C00089__DRA**	Sucrose	-0.616084
**EX_C00244__DRA**	Nitrate	-0.520394
**EX_C00059__DRA**	Sulfate	-0.016141
**EX_C00009__DRA**	Orthophosphate	-0.007504
**EX_C00305__DRA**	Magnesium cation	-2e-06
**EX_C14818__DRA**	$Fe2^+$	-1e-06
**EX_C00237__DRA**	CO	2e-06
**EX_C00288__DRA**	$HCO3^-$	5e-06
**EX_BIOMASS__CYTO**	e-Biomass	0.11
**EX_C00011__DRA**	CO_2	3.156341
**EX_C00001__DRA**	H_2O	3.959216

Table C Summary of a pFBA applied to the leaf model in photorespiratory conditions. The biomass was fixed to 0.11 /h and the objective function was defined as the minimization of photon uptake. The carboxylation/oxygenation ratio of Rubisco was fixed to 3:1.

REACTION	METABOLITE	FLUX
**EX_C00205__DRA**	hn	-74.164666
**EX_C00011__DRA**	CO2	-4.236668
**EX_C00001__DRA**	H2O	-2.817708
**EX_C00244__DRA**	Nitrate	-0.520394
**EX_C00059__DRA**	Sulfate	-0.016141
**EX_C00009__DRA**	Orthophosphate	-0.007504
**EX_C00080__DRA**	H^+	-5e-06
**EX_C00305__DRA**	Magnesium cation	-2e-06
**EX_C14818__DRA**	$Fe2^+$	-1e-06
**EX_C00237__DRA**	CO	2e-06
**EX_BIOMASS__CYTO**	e-Biomass	0.11
**EX_C00007__DRA**	Oxygen	5.582437

Table D Summary of a pFBA applied to the inner bark in heterotrophic conditions. The maximum uptake of amino acids and sucrose was fixed to 1 mmol/gDW/h), while maximizing the biomass production.

REACTION	METABOLITE	FLUX
**EX_C00007__DRA**	Oxygen	-22.697857
**EX_C00049__DRA**	L-Aspartate	-1.0
**EX_C00062__DRA**	L-Arginine	-1.0
**EX_C00082__DRA**	L-Tyrosine	-1.0
**EX_C00079__DRA**	L-Phenylalanine	-1.0
**EX_C00078__DRA**	L-Tryptophan	-1.0
**EX_C00152__DRA**	L-Asparagine	-1.0
**EX_C00065__DRA**	L-Serine	-1.0
**EX_C00064__DRA**	L-Glutamine	-1.0
**EX_C00089__DRA**	Sucrose	-1.0
**EX_C00041__DRA**	L-Alanine	-1.0
**EX_C00037__DRA**	Glycine	-1.0
**EX_C00025__DRA**	L-Glutamate	-1.0
**EX_C00188__DRA**	L-Threonine	-1.0
**EX_C00123__DRA**	L-Leucine	-0.078247
**EX_C00183__DRA**	L-Valine	-0.052161
**EX_C00047__DRA**	L-Lysine	-0.046087
**EX_C00407__DRA**	L-Isoleucine	-0.042019
**EX_C00135__DRA**	L-Histidine	-0.021498
**EX_C00073__DRA**	L-Methionine	-0.019969
**EX_C00009__DRA**	Orthophosphate	-0.01663
**EX_C00059__DRA**	Sulfate	-0.015802
**EX_C14818__DRA**	$Fe2^{+}$	-2e-06
**EX_C00237__DRA**	CO	4e-06
**EX_C00288__DRA**	$HCO3^{-}$	1.5e-05
**EX_BIOMASS__CYTO**	e-Biomass	0.928515
**EX_C00086__DRA**	Urea	0.949947
**EX_C00027__DRA**	Hydrogen peroxide	0.966568
**EX_C00954__DRA**	Indole-3-acetate	0.987862
**EX_C00001__DRA**	H2O	10.983085
**EX_C00014__DRA**	Ammonia	14.308021
**EX_C00011__DRA**	CO2	26.846656

Table E Summary of a pFBA applied to the virgin phellogen model in heterotrophic conditions. The maximum uptake of amino acids and sucrose was fixed to 1 mmol/gDW/h, while maximizing the biomass production.

REACTION	METABOLITE	FLUX
**EX_C00007__DRA**	Oxygen	-19.291057
**EX_C00049__DRA**	L-Aspartate	-1.0
**EX_C00065__DRA**	L-Serine	-1.0
**EX_C00082__DRA**	L-Tyrosine	-1.0
**EX_C00079__DRA**	L-Phenylalanine	-1.0
**EX_C00188__DRA**	L-Threonine	-1.0
**EX_C00025__DRA**	L-Glutamate	-1.0
**EX_C00078__DRA**	L-Tryptophan	-1.0
**EX_C00037__DRA**	Glycine	-1.0
**EX_C00041__DRA**	L-Alanine	-1.0
**EX_C00047__DRA**	L-Lysine	-1.0
**EX_C00089__DRA**	Sucrose	-1.0
**EX_C00152__DRA**	L-Asparagine	-1.0
**EX_C00062__DRA**	L-Arginine	-1.0
**EX_C00064__DRA**	L-Glutamine	-1.0
**EX_C00123__DRA**	L-Leucine	-0.079949
**EX_C00009__DRA**	Orthophosphate	-0.066028
**EX_C00183__DRA**	L-Valine	-0.053295
**EX_C00407__DRA**	L-Isoleucine	-0.042933
**EX_C00135__DRA**	L-Histidine	-0.021965
**EX_C00073__DRA**	L-Methionine	-0.020404
**EX_C00059__DRA**	Sulfate	-0.016471
**EX_C14818__DRA**	$Fe2^+$	-2e-06
**EX_C00237__DRA**	CO	4e-06
**EX_C00080__DRA**	H^+	0.010756
**EX_C00027__DRA**	Hydrogen peroxide	0.088087
**EX_BIOMASS__CYTO**	e-Biomass	0.859321
**EX_C00086__DRA**	Urea	0.948858
**EX_C00322__DRA**	2-Oxoadipate	0.952911
**EX_C00954__DRA**	Indole-3-acetate	0.987593
**EX_C00001__DRA**	H2O	10.17163
**EX_C00014__DRA**	Ammonia	16.196968
**EX_C00011__DRA**	CO2	28.032917

Table F Summary of a pFBA applied to the reproduction phellogen model in heterotrophic conditions. The maximum uptake of amino acids and sucrose was fixed to 1 mmol/gDW/h while maximizing the biomass production.

REACTION	METABOLITE	FLUX
**EX_C00007__DRA**	Oxygen	-18.831028
**EX_C00049__DRA**	L-Aspartate	-1.0
**EX_C00064__DRA**	L-Glutamine	-1.0
**EX_C00082__DRA**	L-Tyrosine	-1.0
**EX_C00079__DRA**	L-Phenylalanine	-1.0
**EX_C00188__DRA**	L-Threonine	-1.0
**EX_C00025__DRA**	L-Glutamate	-1.0
**EX_C00078__DRA**	L-Tryptophan	-1.0
**EX_C00037__DRA**	Glycine	-1.0
**EX_C00041__DRA**	L-Alanine	-1.0
**EX_C00152__DRA**	L-Asparagine	-1.0
**EX_C00089__DRA**	Sucrose	-1.0
**EX_C00065__DRA**	L-Serine	-1.0
**EX_C00062__DRA**	L-Arginine	-1.0
**EX_C00123__DRA**	L-Leucine	-0.078287
**EX_C00009__DRA**	Orthophosphate	-0.064656
**EX_C00183__DRA**	L-Valine	-0.052188
**EX_C00047__DRA**	L-Lysine	-0.04611
**EX_C00407__DRA**	L-Isoleucine	-0.042041
**EX_C00135__DRA**	L-Histidine	-0.021509
**EX_C00073__DRA**	L-Methionine	-0.01998
**EX_C00059__DRA**	Sulfate	-0.016128
**EX_C14818__DRA**	$Fe2^{+}$	-2e-06
**EX_C00237__DRA**	CO	4e-06
**EX_C00080__DRA**	H^{+}	0.010532
**EX_C00027__DRA**	Hydrogen peroxide	0.107041
**EX_BIOMASS__CYTO**	e-Biomass	0.841461
**EX_C00086__DRA**	Urea	0.949921
**EX_C00954__DRA**	Indole-3-acetate	0.987851
**EX_C00001__DRA**	H2O	10.70845
**EX_C00014__DRA**	Ammonia	14.305879
**EX_C00011__DRA**	CO2	28.82203
